# Supplementary material for: Distinct gene expression signatures comparing latent tuberculosis infection with different routes of Bacillus Calmette-Guérin vaccination
Source: Nat Commun. 2023 Dec 21;14:8507. doi: 10.1038/s41467-023-44136-8 (PMC10739751; doi:10.1038/s41467-023-44136-8)
Supplement: Supplementary file 1 — Supplementary Information [file 41467_2023_44136_MOESM1_ESM.pdf]

Supplementary Figure 1

Blood: PCA of Naive Controls and LTBI +/- BCG

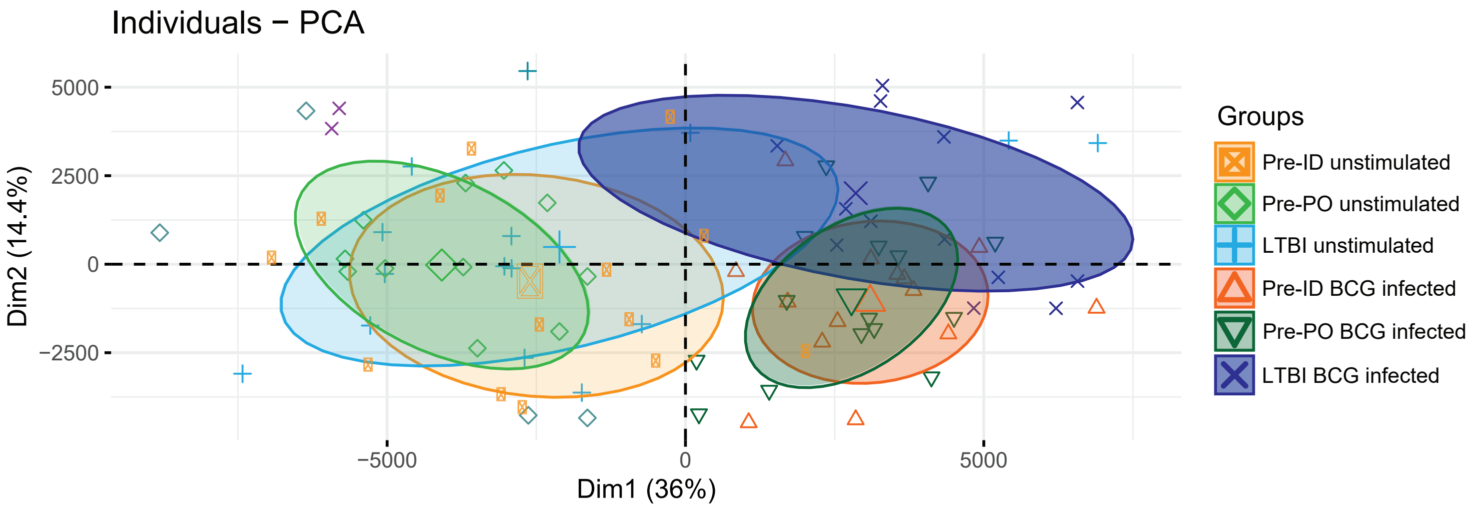

Supplementary Figure 1. Principal component analysis (PCA) of absolute expression data (gene CPMS) of co-cultured peripheral blood CD4+ T cells and autologous MDDC. As displayed in terms of Dimension 1 (y-axis) and Dimension 2 (x-axis), the blood CD4+ T cell gene expression profiles of both pre-ID BCG volunteers and pre-PO-BCG volunteers show substantial overlap at baseline (light orange and light green ovals, respectively) and following in vitro BCG infection (dark orange and dark green ovals), supporting the validity of combining these two sets of volunteers into a single “Mtb/BCG-naïve” control group. In contrast, the gene expression profile of LTBI individuals is substantially different from the pre-vaccinated volunteers both at baseline (light blue) and following in vitro BCG stimulation (dark blue).

Supplementary Figure 2

a. Blood Baseline Genes

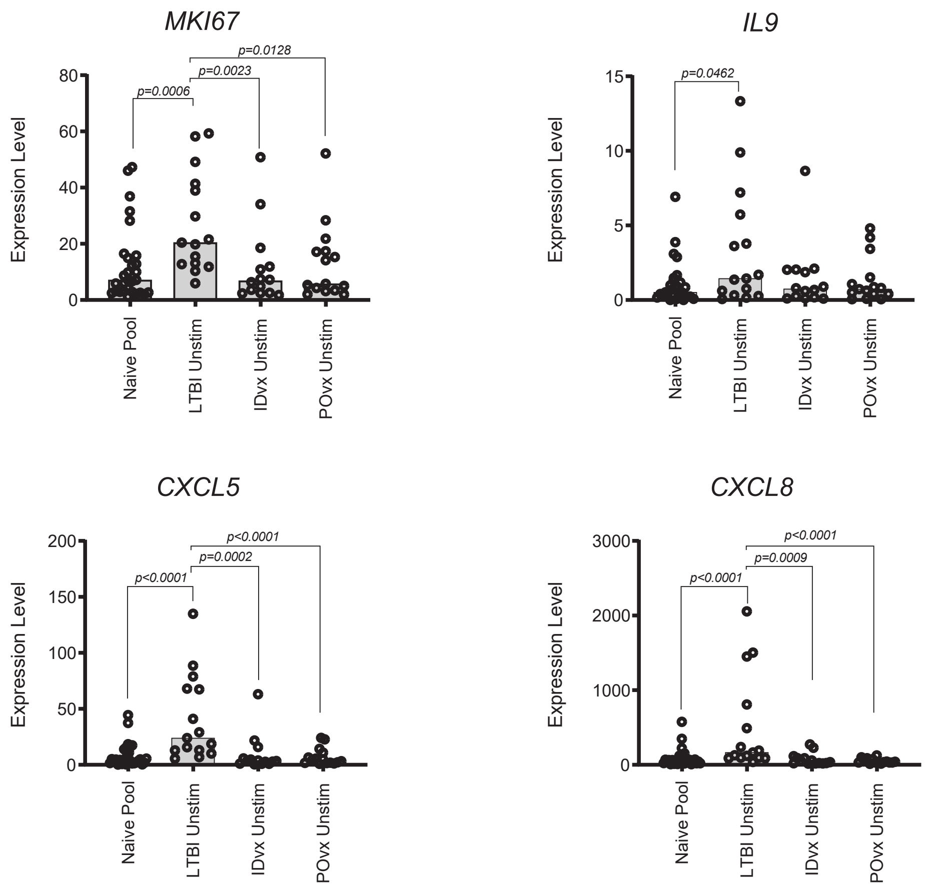

b. BAL Baseline Genes

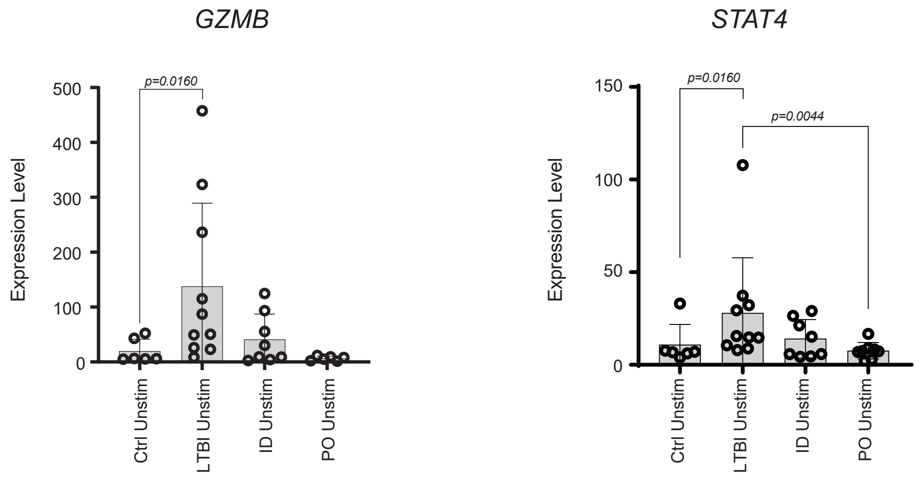

Supplementary Figure 2. Expression of representative genes contributing to baseline immune activation in blood and BAL of LTBI individuals. Baseline blood CD4+ T cell gene expression of MKI67, IL9, CXCL5, and CXCL8 provide examples of significant ongoing immune responses that are observed in LTBI individuals (n=10), but not in the combined (pre-vaccination) naïve pool (n=29) or in recipients of ID or PD BCG (n=14 and n=15, respectively, a). Similarly, significantly increased baseline BAL responses were detected in GZMB and STAT4 in LTBI individuals (n=10) compared to the dedicated Mtb/BCG-naïve volunteers (n=6) and to recipients of ID as well as PO+/-ID BCG (n=8 in each case, b). For all graphs, bars represent mean values and SD; p-values were determined based on two-tailed Mann-Whitney testing.

Supplementary Figure 3: Blood ΔΔ CompBio

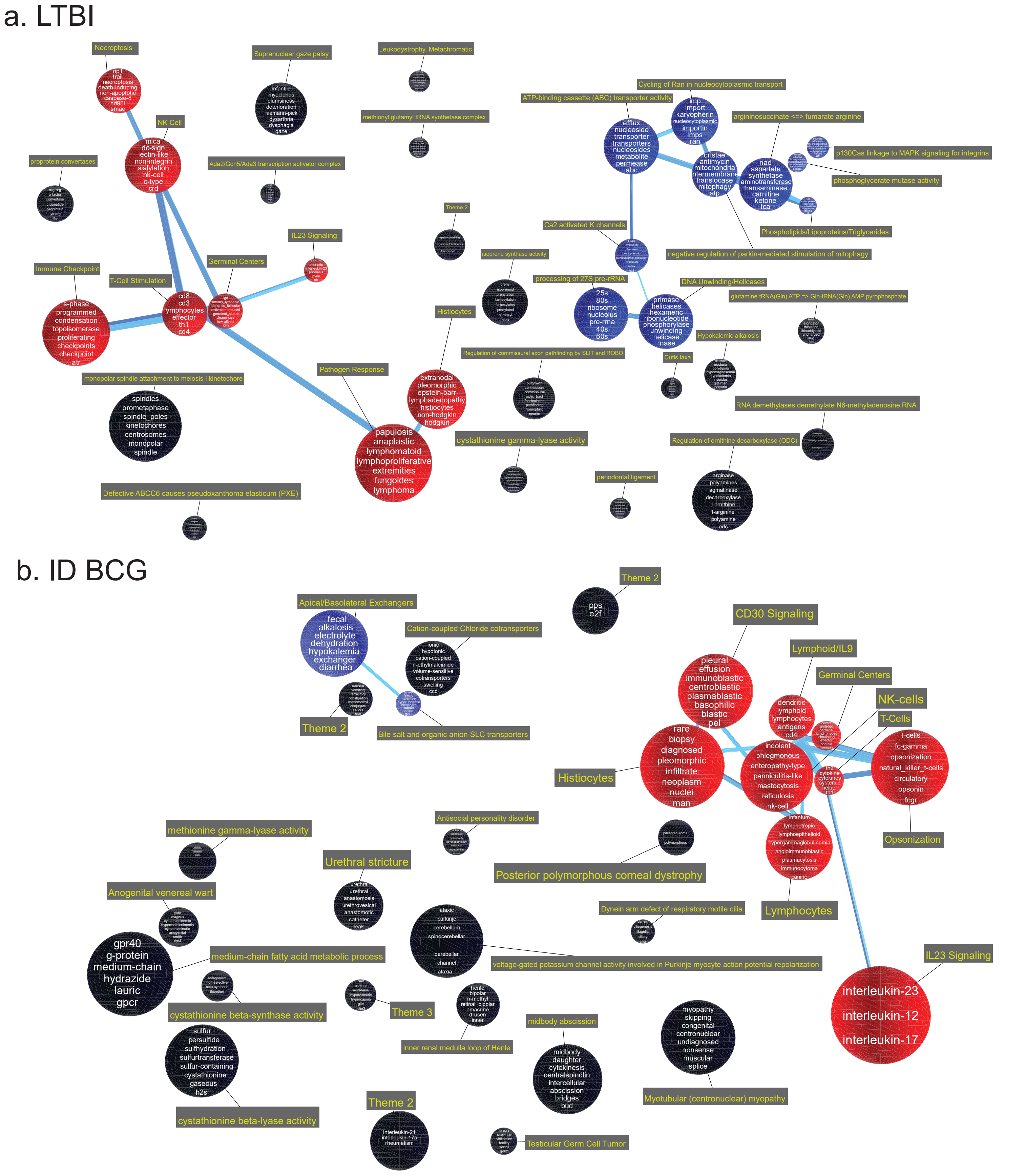

Supplementary Figure 3. Expanded CompBio maps of blood CD4 responses. All significantly enriched themes in the ΔΔ gene expression of blood CD4+ T cells (co-cultured with autologous MDDC) in both LTBI and ID BCG groups are displayed (Supplementary Figure panels 3a and 3b, respectively). In addition to immune response themes detailed further in Figure 3 (indicated by red spheres), LTBI individuals and recipients of ID BCG each display another interconnected cluster of non-immune themes (represented by blue spheres). Additional themes observed in both study groups showed no interconnections and are therefore displayed as black spheres.

Supplementary Figure 4: Blood Δ

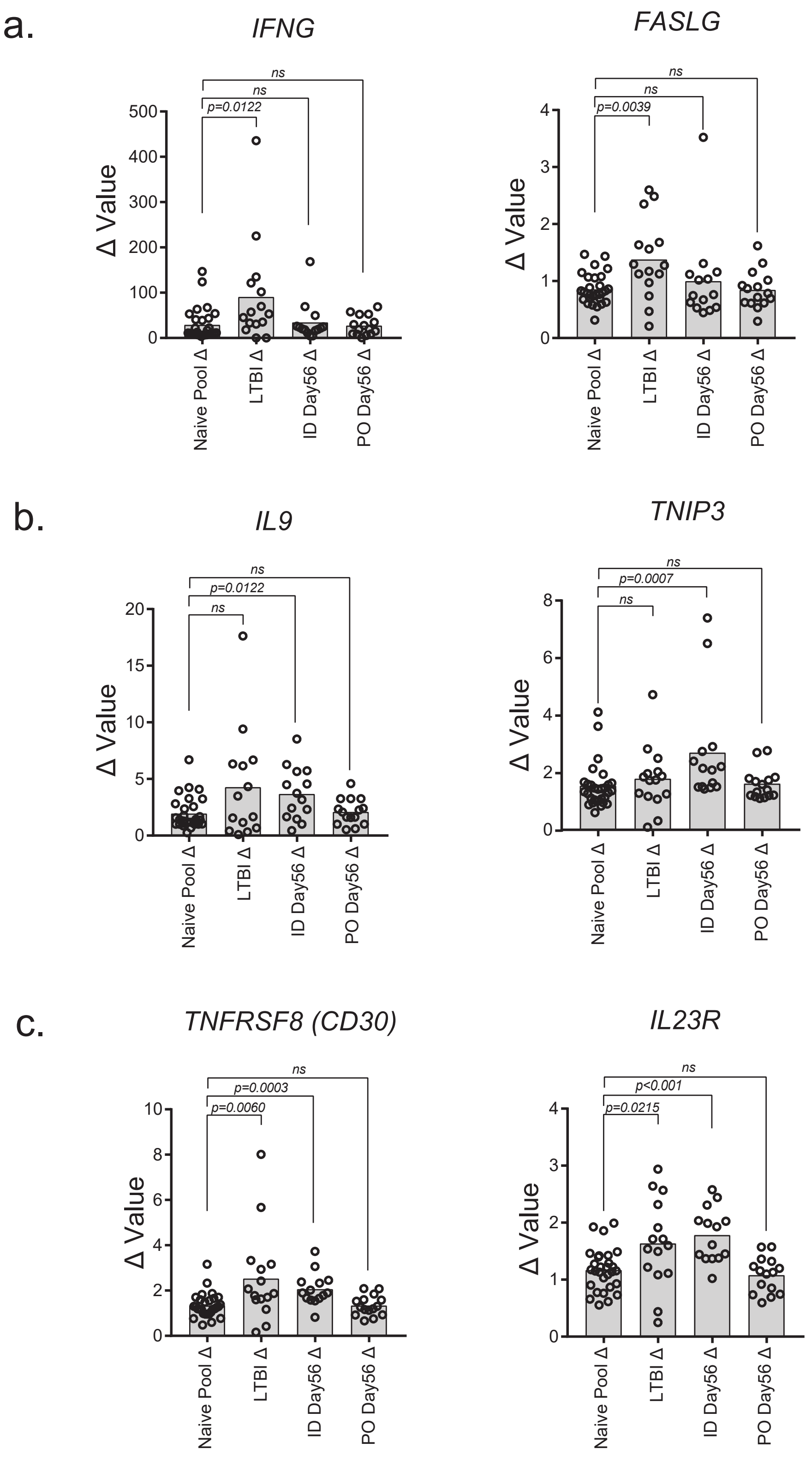

Supplementary Figure 4. Examples of genes induced by in vitro BCG stimulation in peripheral blood cells from LTBI individuals and recipients of ID BCG. Typical peripheral blood gene expression patterns across all study groups are presented in graphic form. As illustrated in panel 4a, cultured blood CD4+ T cells from LTBI individuals uniquely demonstrated significant increases in expression of IFNG and FASLG following in vitro stimulation despite the higher baseline expression of these genes, whereas panel 4b shows that IL9 and TNIP3 genes were most strongly upregulated in blood CD4+ T cell responses in recipients of ID BCG. Overlap was also observed, as illustrated in panel 4c for TNFRSF8 and IL23R upregulated gene expression levels detected in both LTBI individuals and recipients of PO BCG. For all graphs, bars again display mean and SD values; statistical assessment utilized two-tailed Mann-Whitney tests. For all blood studies (4a), n=29 for the naïve pool, n=15 for LTBI, n=14 for ID BCG, and n=15 for PO BCG. For BAL studies (4b), n=6 naïve, n=10 LTBI, and n=8 for both ID and PO±ID BCG groups.

Supplementary Figure 5: BAL ΔΔ CompBio

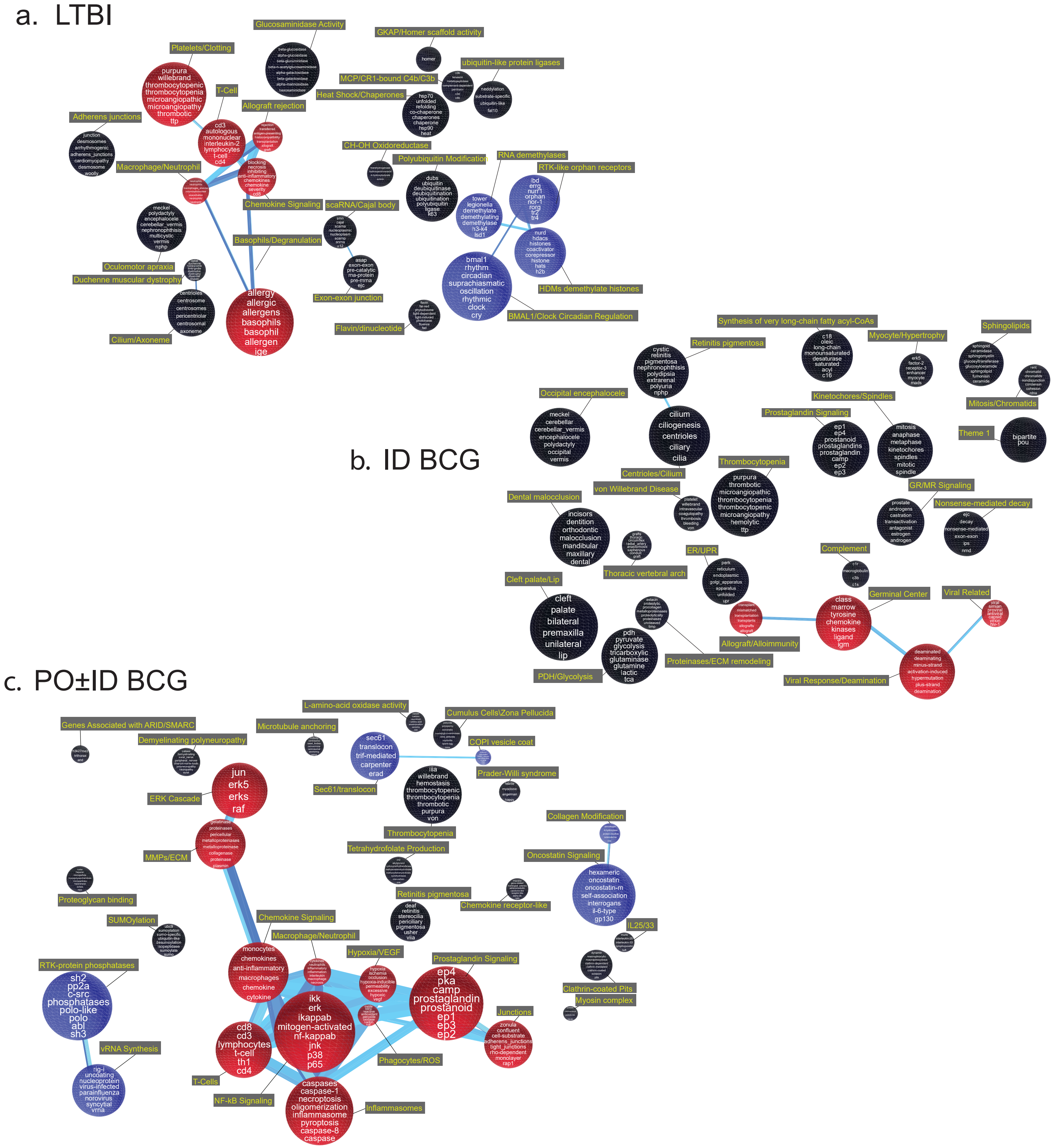

Supplementary Figure 5. Expanded CompBio map findings in the ΔΔ gene analyses of BAL cells from LTBI, ID BCG and PO BCG. Full maps of themes identified by CompBio analysis are displayed for studies of BAL cells from individuals with LTBI (panel 5a), and recipients of ID or PO±ID BCG (panels 5b and 5c, respectively). Red spheres again indicate interconnected immune themes for all study groups, whereas interconnected non-immune themes are displayed as blue spheres. Black spheres indicate non-interconnected themes. The maps emphasize that, compared to the other study groups, BAL cells from recipients of ID BCG display a more limited array of both immune-associated themes and of interrelated themes more generally. In contrast, BAL cells from both LTBI individuals and recipients of PO±ID BCG display complex networks of interconnected immune themes as well as interconnected non-immune themes.

Supplementary Figure 6: BAL Δ

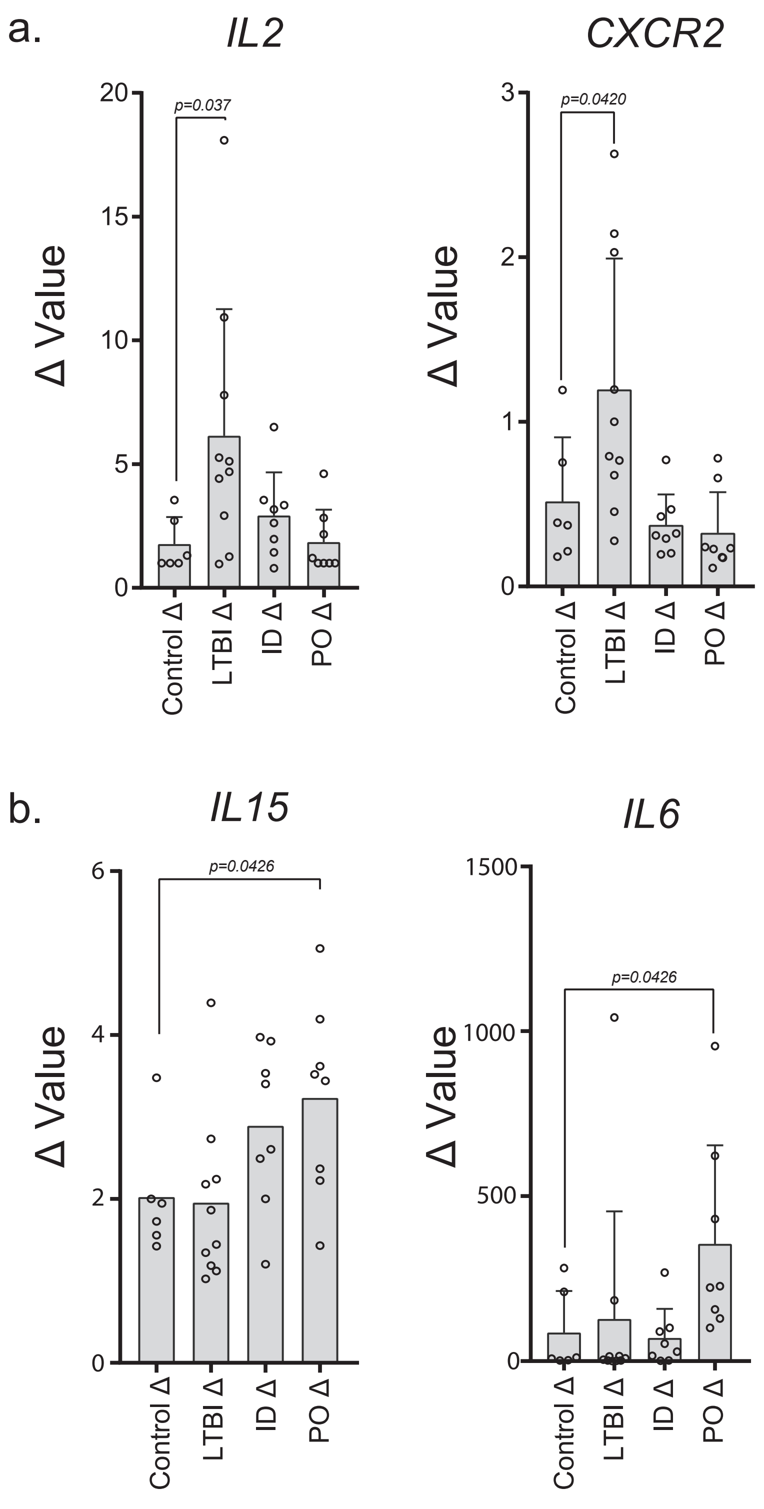

Supplementary Figure 6. Selected Mtb-induced gene expression patterns in BAL from all groups for specific genes prominently expressed by LTBI and BCG-vaccinated individuals. For studies of BAL cells, genes associated with T cell stimulation were upregulated in both the LTBI and PO±ID BCG study groups; however, the genes that contributed to identification of this theme were not the same for the two groups. Mtb-induced expression of IL2 and CXCR2 (panel a) was significant only in LTBI individuals despite their higher baseline expression in these volunteers. In contrast, recipients of PO±ID BCG uniquely displayed significant increases in Mtb-induced induction of IL15 and IL6 (panel b). Assessment of p-values utilized two-tailed Mann-Whitney tests. Bars represent mean and SD values. For all graphs, n=6 controls, n=10 LTBI, and n=8 for both ID and PO±ID BCG groups.

Supplementary Figure 7

BAL Expression of *DPP4 (CD26)*

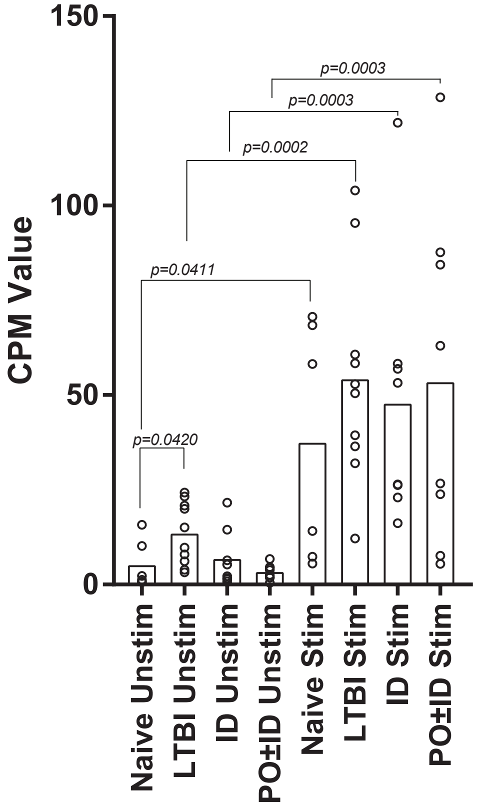

Supplementary Figure 7. Baseline and Mtb-induced BAL cell expression of MAIT cell associated marker DPP4 (CD26). CD26 is a selective (but not specific) marker of MAIT cell activation. DPP4 was uniquely increased in LTBI individuals compared to Mtb/BCG naïve volunteers at baseline. Following in vitro Mtb infection, DPP4 expression increased in BAL cells of naïve volunteers; increases in LTBI both BCG vaccinated groups was observed as well, with greater statistical significance. Bars display mean and SD values; p-values were determined using two-tailed Mann-Whitney tests. Results are shown for n=6 controls, n=10 LTBI individuals, and n=8 for both ID and PO±ID BCG groups.

Supplementary Table I. Demographics and BAL Subsets

Supplementary Table I: Participant demographics and BAL parameters. A: In the PBMC substudy, the pre-vaccination samples of all 29 participants who later received BCG vaccination were pooled to create a single Mtb/BCG-naïve control group. As indicated, further blood samples were collected 56 days (0.15 years) after vaccination with ID or PO BCG. In contrast, blood samples were collected an average of over 6 years after the diagnosis of LTBI (IA). **B:** As no BAL samples were collected before protocol vaccination or diagnosis of LTBI, a separate group of Mtb/BCG-naïve volunteers were recruited for participation in the BAL sub-study, along with LTBI individuals and subsets of study vaccine recipients who fit eligibility criteria and were agreeable to research bronchoscopy participation. Vaccine recipients underwent research bronchoscopy procedures an average of 4.25 (+/- 3.15) years following ID BCG and 7.75 (+/- 5.09) years after PO BCG (N/S). **C:** BAL cell profiles demonstrated no significant differences between the four study groups with regard to total BAL cell counts. Differential BAL cell counts of Mtb-naïve and LTBI individuals displayed no significant differences; however, BAL of recipients of ID BCG alone displayed a significantly lower percentage of alveolar macrophages (AM) than that of both Mtb/BCG-naïve controls (+, p=0.006) and LTBI individuals (*, p=0.031); a corresponding increase in BAL lymphocyte percentage in ID BGC recipients was also significant in comparison to the LTBI group (*, p=0.031) but not in comparison to naïve controls. The percentage of BAL AM in recipients of PO±ID BCG vaccination also displayed significantly lower percentage of AM than individuals with LTBI (*, p= 0.034); this percentage was not significantly different from than that observed in Mtb/BCG-naïve volunteers or in recipients of ID BCG alone. In mixed cell samples such as BAL, transcriptional data alone cannot distinguish whether the increased transcript abundance between study groups is due to upregulation of expression, difference in cellular composition with constant expression per cell, or a combination thereof. We therefore analyzed whether observed differences in gene expression showed correlation with the BAL differential cells counts; because no correlation was found we did not include correction for these counts in our subsequent analysis.

Subject groups	**Mtb/BCG-naive**	**LTBI**	**ID BCG**	**PO±ID BCG**
**a. DEMOGRAPHICS—PBMC substudy**				
Samples analyzed (n)	29	15	14	15
Age, years (mean, SD)	31.72 (+/-6.32)	29.7 (+/- 6.76)	31.50 (+/-6.70)	31.93 (+/-6.18)
Male gender (%)	34	47	36	33
Years since LTBI diagnosis or last BCG vaccine dose	n/a	6.13 (+/-4.93)	0.15 (+/-0)	0.15 (+/-0)
**b. DEMOGRAPHICS—BAL cell substudy**				
Samples analyzed (n)	6	10	8	8
Age, years (mean, SD)	26.0 (+/-5.18)	29.7 (+/-6.07)	36.0 (+/-8.45)	38.88 (+/-8.04)
Male gender (%)	50	60	50	12.5
Years since LTBI diagnosis or last BCG vaccine dose	n/a	9.20 (+/-6.41)	4.25 (+/-3.15)	7.75 (+/-5.09)
**c. BAL PARAMETERS**				
Total cell count	1.57e7 (+/-19.5e6)	1.43e7 (+/-7.70e6)	1.43e7 (+/-1.21e7)	1.07e7 (+/-3.70e6)
Differential counts (%)				
macrophages	95.44 (+/-3.37)	95.54 (+/-2.26)	+,* 88.4 (+/-6.66)	* 92.71 (+/-2.79)
lymphocytes	3.11 (+/-3.40)	3.86 (+/-2.37)	* 9.29 (+/-6.73)	5.25 (+/-3.02)
neutrophils	1.33 (+/-1.19)	0.50 (+/-0.50)	1.90 (+/-2.92)	1.96 (+/-3.55)
eosinophils	0.11 (+/-0.17)	0.07(+/-0.14)	0.43 (+/-0.53)	0.08 (+/-0.15)

+ p<0.01 in comparison to naïve subjects; * p<0.05 compared to LTBI subjects

Supplementary Table II. Cytokine Protein Responses

Supplementary Table II: Protein-level assessment of CD4+ T cell responses in blood and BAL from LTBI individuals and recipients of ID and PO BCG. Supernatant concentrations of IFN-Y, TNF, IL-2 and IL-9 (in pg/mL) are presented for from CD4+ T cells co-cultured with autologous MDDC incubated in medium alone and infected with BCG at MOI of 20 (IIa), and unsorted BAL cells following overnight incubation in medium or infection with Mtb (MOI 3:1), (IIb). Because of the non-parametric nature of the findings, results are expressed as medians and ranges for each study group. Stimulated responses were compared with unstimulated responses ("Δ" comparisons) in each group by Wilcoxon matched pairs assessment. Stimulated responses for each experimental group were compared with stimulated responses in the control groups ("ΔΔ" responses) by Mann-Whitney U tests. Blood and BAL experimental and control groups were also analyzed by ANOVA Kruskal/Wallis post-hoc tests. Baseline production of these cytokines in medium alone was minimal for all study groups for both blood CD4+ T cells and BAL cells. Blood CD4+ T cells from each group showed significant BCG-induced production of IFN-Y, TNF and IL-2. In contrast, only blood from LTBI individuals and ID BCG recipients displayed significant IL-9 production following BCG stimulation (by Wilcoxon matched pairs analysis, $p<0.05$). In comparison to cytokine induction in the Mtb/BCG-naïve control group, significant BCG-induced production of IFN-Y was seen only in LTBI individuals, whereas TNF, IL-2, and IL-9 responses were significant for both LTBI participants and ID BCG recipients. Recipients of PO BCG showed no significant blood CD4+ T cell responses in any ΔΔ comparisons (by Mann-Whitney, $p<0.05$). By Kruskal/Wallis ANOVA, BCG-induced production of IFNY, IL-2 and TNF was significantly increased only for LTBI individuals, whereas IL-9 induction remained significant for both recipients of ID BCG and the LTBI group. In BAL cell studies, when compared to Mtb-induced cytokine production in Mtb/BCG-naïve volunteers, increases in IFN-Y and IL-2 were significant only for LTBI individuals, whereas TNF production was significant in LTBI and PO±ID BCG groups. Mtb-induced increases in IL-9 were significant only in recipients of PO BCG (Mann-Whitney, $p<0.05$). Of these responses, increased TNF production remained significantly increased in the Kruskal-Wallis/ANOVA analysis only in recipients of PO±ID BCG. All statistical tests used two-tailed analysis.

a. Blood

					BCG/Medium	Group BCG/Naïve poo lBCG	
	Group	Stimulation	Median	Range(Min-Max)	P by Wilcoxon test	P by Mann-Whitney test	P by ANOVA Kruskal-Wallis test
IFN-γ	Naïve	BCG	1490	0-4060			
		Medium	20.01	1.3-502	***<0.0001***		
	LTBI	BCG	4454	6.3-5404			
		Medium	31.27	0-213	***<0.0001***	***0.0074***	***0.014***
	ID	BCG	2409	77-4233			
		Medium	40.53	1.2-307	***0.0001***	0.1606	0.8021
	PO	BCG	1827	159-3853			
		Medium	23.91	2.7-117	***0.0005***	0.7594	>0.9999
TNF	Naïve	BCG	96.97	0.7-657			
		Medium	3.45	0-93	***<0.0001***		
	LTBI	BCG	1146	0.4-7999			
		Medium	7.49	1.1-64	***<0.0001***	***0.0064***	***0.0087***
	ID	BCG	237.3	31-1028			
		Medium	4.12	0-41	***0.0001***	***0.0171***	0.1507
	PO	BCG	50.68	10-428			
		Medium	2.825	0-7	***0.0005***	0.6534	>0.9999
IL-2	Naïve	BCG	1099	2.6-5300			
		Medium	124.7	3.3-1605	***<0.0001***		
	LTBI	BCG	4088	5.3-11843			
		Medium	79.23	0-699	***<0.0001***	***0.0185***	***0.0165***
	ID	BCG	2974	646-6777			
		Medium	170.7	1.9-1132	***0.0001***	***0.0113***	0.076
	PO	BCG	1496	451-5300			
		Medium	169.9	19.9-337	***0.0005***	0.2546	>0.9999
IL-9	Naïve	BCG	0	0-5.3			
		Medium	0	0-5.5	0.0781		
	LTBI	BCG	6.22	0-26.6			
		Medium	0	0-6.6	***0.001***	***0.0081***	***0.0132***
	ID	BCG	3.51	0-16.2			
		Medium	0	0-5.3	***0.0098***	***0.0083***	***0.0369***
	PO	BCG	0	0-3.7			
		Medium	0	0-4.0	0.1563	0.4858	>0.9999

b. BAL

					Mtb/Medium	Group Mtb/Naïve Mtb	
	Group	Stimulation	Median	Range(Min-Max)	P by Wilcoxon test	P by Mann-Whitney test	P by ANOVA Kruskal-Wallis test
IFN-γ	Naïve	Mtb	94.87	1.4-175			
		Medium	1.645	0-80	***0.0005***		
	LTBI	Mtb	279.9	1.4-2111			
		Medium	2485	0.2-124	***0.001***	***0.0449***	0.1039
	ID	Mtb	107.6	2.0-1784			
		Medium	0.77	0-187	0.1094	0.5918	>0.9999
	PO±ID	Mtb	142.5	3.2-436			
		Medium	0.84	0-1.5	***0.0039***	0.2773	0.9026
TNF	Naïve	Mtb	4513	47-22800			
		Medium	1633	14-15578	0.5186		
	LTBI	Mtb	10576	618-20956			
		Medium	590	20-19504	***0.0034***	***0.0196***	0.0834
	ID	Mtb	6216	2811-7256			
		Medium	25.83	0-21989	0.2959	0.6951	>0.9999
	PO±ID	Mtb	17116	2235-21466			
		Medium	25.52	8.5-258	***0.0039***	***0.0052***	0.0037
IL-2	Naïve	Mtb	58.54	0.3-335			
		Medium	0.86	0.0-24	***0.005***		
	LTBI	Mtb	227.1	21-1804			
		Medium	0.625	0.0-144	***0.005***	***0.0296***	0.0872
	ID	Mtb	109.7	0.3-1949			
		Medium	0.26	0.0-150	0.1563	0.4824	>0.9999
	PO±ID	Mtb	147.3	2.0-452			
		Medium	0.07	0.0-0.8	***0.0039***	0.5079	>0.9999
IL-9	Naïve	Mtb	0.008	0.00-0.01			
		Medium	0.007	0.00-0.01	0.8223		
	LTBI	Mtb	0.0085	0.00-0.02			
		Medium	0.009	0.00-0.02	0.1812	0.3496	>0.9999
	ID	Mtb	0.007	0.00-0.02			
		Medium	0.007	0.00-0.01	0.9844	0.8305	>0.9999
	PO±ID	Mtb	0.01	0.00-0.01			
		Medium	0.007	0.00-0.01	0.0625	***0.0163***	0.0924

Supplementary Table III.

Supplementary Table III: Numbers of DEGs identified in Δ and ΔΔ comparisons. The table displays the numbers of differentially-expressed genes (DEG) in studies of BCG stimulation of blood CD4+ T cell /autologous MDDC (a) and by Mtb infection of unsorted BAL cells (b). For each arm of the study, Δ comparisons indicate the numbers of DEGs identified as differentially-expressed in infected vs. uninfected cells of each study group (left). For ΔΔ comparisons, DEGs reflect differences in infection-induced gene expression in each study group as compared to that observed in Mtb/BCG naïve cohorts of each study arm (right). As indicated in Figure 1 of the main text, for blood studies, n=29 for the naïve pool, n=15 for LTBI, n=14 for ID BCG, and n=15 for PO BCG. For BAL studies, n=6 naïve, n=10 LTBI, and n=8 for both ID and PO±ID BCG groups.

a. Blood Gene Lists

Blood Δ

Control	Up	512
Control	Down	812
LTBI	Up	464
LTBI	Down	30
ID	Up	473
ID	Down	920
PO	Up	499
PO	Down	673

Blood ΔΔ

LTBI	Up	212
LTBI	Down	187
ID	Up	38
ID	Down	78
PO	Up	28
PO	Down	30

b. BAL Gene Lists

BAL Δ

Control	Up	295
Control	Down	314
LTBI	Up	625
LTBI	Down	592
ID	Up	977
ID	Down	1362
PO	Up	1308
PO	Down	2349

BAL ΔΔ

LTBI	Up	108
LTBI	Down	173
ID	Up	97
ID	Down	241
PO	Up	324
PO	Down	1083
