# Supplementary material for: Distinct gene expression signatures comparing latent tuberculosis infection with different routes of Bacillus Calmette-Guérin vaccination
Source: Nat Commun. 2023 Dec 21;14:8507. doi: 10.1038/s41467-023-44136-8 (PMC10739751; doi:10.1038/s41467-023-44136-8)
Supplement: Supplementary file 3 — Reporting Summary [file 41467_2023_44136_MOESM3_ESM.pdf]

nature portfolio

Corresponding author(s): Richard F. Silver, M.D.
Daniel F. Hoft, M.D., Ph.D.

Last updated by author(s): 10/23/2023

Reporting Summary

Nature Portfolio wishes to improve the reproducibility of the work that we publish. This form provides structure for consistency and transparency in reporting. For further information on Nature Portfolio policies, see our Editorial Policies and the Editorial Policy Checklist.

Statistics

For all statistical analyses, confirm that the following items are present in the figure legend, table legend, main text, or Methods section.

n/a	Confirmed	
☐	☒	The exact sample size (*n*) for each experimental group/condition, given as a discrete number and unit of measurement
☐	☒	A statement on whether measurements were taken from distinct samples or whether the same sample was measured repeatedly
☐	☒	The statistical test(s) used AND whether they are one- or two-sided<br>*Only common tests should be described solely by name; describe more complex techniques in the Methods section.*
☐	☒	A description of all covariates tested
☐	☒	A description of any assumptions or corrections, such as tests of normality and adjustment for multiple comparisons
☒	☐	A full description of the statistical parameters including central tendency (e.g. means) or other basic estimates (e.g. regression coefficient) AND variation (e.g. standard deviation) or associated estimates of uncertainty (e.g. confidence intervals)
☐	☒	For null hypothesis testing, the test statistic (e.g. *F*, *t*, *r*) with confidence intervals, effect sizes, degrees of freedom and *P* value noted<br>*Give P values as exact values whenever suitable.*
☒	☐	For Bayesian analysis, information on the choice of priors and Markov chain Monte Carlo settings
☒	☐	For hierarchical and complex designs, identification of the appropriate level for tests and full reporting of outcomes
☒	☐	Estimates of effect sizes (e.g. Cohen's *d*, Pearson's *r*), indicating how they were calculated

Our web collection on statistics for biologists contains articles on many of the points above.

Software and code

Policy information about availability of computer code

Data collection	No private or custom code was used for data collection
Data analysis	No private code was used in this analysis; all software used was either publicly or commercially available. RNAseq data were processed using STAR (v 2.0.4b) for alignment with Ensembl release 76 top-level assembly. Counts were derived using Subread:featureCount (v1.4.5) and quality assessment was performed using RSeQC (v2.3). The R (v3.4.1)/BioConductor packages EdgeR (v2.20.2) and Limma (v3.34.4) were used to adjust counts for differences in library size. Transcriptomic analysis was performed using CompBio (https://www.percayai.com/). GraphPad Prism (v10.0.3) was used for graphs and calculation of statistical significance. Microsoft Excel (v2308) was used for spreadsheets. Blender (v2.93.0) and GIMP (v2.10.22) were used for custom image creation. Heat maps were generated using the R (v4.2.1) package ComplexHeatmap (v2.16.0). Adobe Illustrator (v27.8.1) was used for final figure creation.”

For manuscripts utilizing custom algorithms or software that are central to the research but not yet described in published literature, software must be made available to editors and reviewers. We strongly encourage code deposition in a community repository (e.g. GitHub). See the Nature Portfolio guidelines for submitting code & software for further information.

Data

Policy information about availability of data

All manuscripts must include a data availability statement. This statement should provide the following information, where applicable:

- Accession codes, unique identifiers, or web links for publicly available datasets
- A description of any restrictions on data availability
- For clinical datasets or third party data, please ensure that the statement adheres to our policy

As is now noted in the manuscript, the RNAseq data generated in this study have been deposited in the Gene Expression Omnibus database under accession codes GSE224055 (Blood) and GSE223999 (BAL) for all study subjects who gave specific consent for genomic data sharing. Source data for all graphs included in Figures and Supplemental Figures is provided within the Supplemental materials as the Graph Source Data file.

Human research participants

Policy information about studies involving human research participants and Sex and Gender in Research.

Reporting on sex and gender	This is reported in subject demographic information (Supplemental Table I).
Population characteristics	Also reported in Supplemental Table I
Recruitment	Recruitment for initial BCG vaccine trials was done by accessing the SLU Center for Vaccine Development list of potential subjects (20,000 people have given us permission to contact them about current or future studies), and advertising locally at SLU, and locally by social and printed media. We always strive to make sure our recruitment efforts enroll proportions of all ethnic/racial groups representative of our community. All who met screening criteria were offered enrollment. Randomization was conducted at enrollment in our vaccination studies.<br>For recruitment of bronchoscopy participants, both subjects from prior applicable BCG studies at SLU and respondents to recruitment fliers at CWRU were screened for basic bronchoscopy eligibility (age 18-50, no history or asthma or other chronic lung disease, current non-smoking status). At CWRU, subjects self-selected to respond to advertising fliers. At SLU, prior BCG recipients in the proper age range and without chart documentation of exclusion criteria were contacted to inquire about their potential interest in participating in these additional procedures.
Ethics oversight	As reported, the study was approved by the IRBs of CWRU/University Hospitals of Cleveland, the Louis Stokes Cleveland VA Medical Center, and Saint Louis University. It is also specifically noted that written informed consent was provided by each subject and that subject compensation was provided.

Note that full information on the approval of the study protocol must also be provided in the manuscript.

Field-specific reporting

Please select the one below that is the best fit for your research. If you are not sure, read the appropriate sections before making your selection.

☒ Life sciences ☐ Behavioural & social sciences ☐ Ecological, evolutionary & environmental sciences

For a reference copy of the document with all sections, see nature.com/documents/nr-reporting-summary-flat.pdf

Life sciences study design

All studies must disclose on these points even when the disclosure is negative.

Sample size	Because of the exploratory nature of this research as well as the difficulty recruiting subjects to participate in this complex protocol involving research bronchoscopy procedures, we did not perform a formal sample size calculation. However, our recruitment goal was to include a minimum of 10 subjects for each experimental group.
Data exclusions	No data was excluded unless analysis revealed concerns for quality control (eg, sample contamination) or were determined by statistical analysis to represent clear outliers
Replication	All studies utilized samples from unique individual subjects accounting for the subject numbers reported in the study design (illustrated in Figure 1) and in subsequent results presentation in other study figures and their legends. Because of limiting amounts of these valuable subject samples and the expense of complicated study assays, single samples were utilized in all cases without performance of technical replicates.
Randomization	The initial assignment of BCG-vaccinated subjects to receive ID vs PO vaccination utilized randomization. For immunologic studies performed specifically for the current study, further randomization could not be performed because subject groups were already determined and no further selection was provided regarding which subjects would undergo further study participation (for example, there were not "bronchoscopy" and "non-bronchoscopy" arms to further studies).

Blinding

Again, blinding was performed in the initial BCG vaccination trials, but only limited blinding was appropriate for the immunologic studies performed specifically for this study. Specfically, samples utilized prior coding by subject group (including vaccination groups) and subject numbers; however, the data collection and analysis were blinded with regard to personal and identifying information of all subjects. Because of the large numbers of different types of samples associated with both the vaccine trials and blood and BAL research studies, we felt that any attempt to re-blind the samples further would provide more risk of mislabeling, that could invalidate comparison of paired samples from individual subjects, than benefit from further blinding.

Reporting for specific materials, systems and methods

We require information from authors about some types of materials, experimental systems and methods used in many studies. Here, indicate whether each material, system or method listed is relevant to your study. If you are not sure if a list item applies to your research, read the appropriate section before selecting a response.

Materials & experimental systems

n/a	Involved in the study	
☐	☒	Antibodies
☒	☐	Eukaryotic cell lines
☒	☐	Palaeontology and archaeology
☒	☐	Animals and other organisms
☐	☒	Clinical data
☒	☐	Dual use research of concern

Methods

n/a	Involved in the study	
☒	☐	ChIP-seq
☐	☒	Flow cytometry
☒	☐	MRI-based neuroimaging

Antibodies

Antibodies used

Cytokine concentrations in supernatants from BCG-stimulated blood CD4+ T cell/MDDC cultures and Mtb-infected BAL cells were measured using cytometric bead array (CBA) assessments of IFNg, TNF, and IL-2 (BD Biosciences, 560484). Concentrations of IL-9 and IL-15 were determined by ELISA (Biolegend 434704 and 435104, respectively).

Validation

All antibodies were provided in commercial CBA and ELISA kits as noted above and were used in accordance with the manufacturers' validated protocols.

Clinical data

Policy information about clinical studies

All manuscripts should comply with the ICMJEguidelines for publication of clinical research and a completedCONSORT checklist must be included with all submissions.

Clinical trial registration

NIH clinical DMID trials (for research BCG vaccination) are referenced within the methods.

Study protocol

Trials as noted above are listed in ClinicalTrials.gov

Data collection

Described in study methods.

Outcomes

Described in study methods.

Flow Cytometry

Plots

Confirm that:

☐ The axis labels state the marker and fluorochrome used (e.g. CD4-FITC).

☐ The axis scales are clearly visible. Include numbers along axes only for bottom left plot of group (a 'group' is an analysis of identical markers).

☐ All plots are contour plots with outliers or pseudocolor plots.

☐ A numerical value for number of cells or percentage (with statistics) is provided.

Methodology

Sample preparation

Describe the sample preparation, detailing the biological source of the cells and any tissue processing steps used.

Instrument

Identify the instrument used for data collection, specifying make and model number.

Software	*Describe the software used to collect and analyze the flow cytometry data. For custom code that has been deposited into a community repository, provide accession details.*
Cell population abundance	*Describe the abundance of the relevant cell populations within post-sort fractions, providing details on the purity of the samples and how it was determined.*
Gating strategy	*Describe the gating strategy used for all relevant experiments, specifying the preliminary FSC/SSC gates of the starting cell population, indicating where boundaries between "positive" and "negative" staining cell populations are defined.*

☐ Tick this box to confirm that a figure exemplifying the gating strategy is provided in the Supplementary Information.
